# Developing guidelines for pet rat housing through expert consultation

**DOI:** 10.1002/vetr.1839

**Published:** 2022-07-16

**Authors:** Vikki Neville, Kristina Hunter, Livia Benato, Michael Mendl, Elizabeth S. Paul

**Affiliations:** ^1^ Bristol Veterinary School University of Bristol Bristol UK; ^2^ City Vets Exeter UK

## Abstract

**Background:**

Pet care guidelines play an important role in ensuring that owners are well informed about good husbandry practices, allowing them to provide the best care for their animals. However, the development of such guidelines is difficult when there is little appropriate empirical evidence on which to base guidelines, as in the case of pet rats. The consultation of multiple experts can help to surmount this challenge.

**Methods:**

We developed a set of guidelines for pet rat housing by consulting with a group of experts, including veterinarians, veterinary nurses, animal welfare scientists and experienced pet rat owners. The consultation involved two rounds of online surveys (*n* = 13) and one online discussion (*n* = 8).

**Results:**

The resulting guidelines cover a broad range of features within pet rat housing, including injury prevention, details of suitable refuges and substrates, and suitable cage sizing. The guidelines may evolve as more information about pet rats comes to light but may nonetheless provide a useful starting point for any future guidelines.

**Conclusions:**

At present, these guidelines may not only be useful for pet rat owners and those advising pet rat owners, such as veterinarians, but may also be useful in the design of housing, including for laboratory rodents.

## INTRODUCTION

Providing pet owners with accurate information about pet care is essential for the welfare of their animals, especially as many owners will actively seek advice on what care to provide.^1^, ^2^ Accurate information is even more valuable when there are welfare concerns about the housing and husbandry of animals, as is the case with many less popular companion species,^3^, ^4^, ^5^, ^6^ including rats.^7^

However, developing good care guidelines for pet rats poses a number of challenges. The first challenge is that while much research has been conducted on the care of captive rats, the focus of this research has largely been on laboratory rats. The conditions under which these animals are kept are usually too standardised and lacking in space and variation to provide much useful information about what constitutes good housing in the more spacious and complex environments that are often provided for pet rats.^8^, ^9^, ^10^ Indeed, housing specifically designed for pet rats has been used to provide and study enhancements to laboratory rat welfare.^11^, ^12^ There is thus subpar evidence on which to base any guidelines. The second challenge is that an individual owner's understanding of what good housing and husbandry entails will likely be influenced by their experiences of and beliefs about the species.^13^, ^14^, ^15^ In the case of rats, this may include factors such as knowledge of rat ecology,^16^, ^17^ familiarity with core welfare principles like the five freedoms,^18^, ^19^ the sorts of husbandry‐related injuries that appear in veterinary practices,^20^, ^21^, ^22^ and observations of rats in their home environment and the sorts of activities and enrichment that attract the most interest.^7^, ^23^

Consultation with multiple experts can help to surmount these challenges; consensus methods are considered to be particularly useful when there is little empirical evidence, and they also capitalise on multiple minds (and experiences) being better than one.^24^, ^25^, ^26^ This approach is very useful in the fields of veterinary and animal welfare science. For example, it has been used in the development of pain scales^27^, ^28^ to identify welfare issues and indicators across a number of species^29^, ^30^, ^31^, ^32^, ^33^ and to develop veterinary guidelines.^34^, ^35^

The aim of this study was to develop guidelines for the housing of pet rats through consultation with a pool of experts, comprising individuals with diverse experiences of rats—experienced pet rat owners, veterinary surgeons and nurses, and laboratory rodent welfare scientists. We broadly followed the Delphi method,^25^, ^36^ in which experts were asked to complete a set of surveys, with the results of each prior survey made available to the experts before completion of the subsequent survey. The surveys were thus designed to progressively focus on the important features of good rat housing to prepare final guidelines for pet rat housing.

## METHODS

### Recruitment of experts

We aimed to recruit a minimum of 10 experts with diverse backgrounds^24^, ^36^ and approached experts who were animal welfare scientists (including those whose research focused on laboratory rodent welfare), veterinarians, veterinary nurses, and experienced (>3 years) pet rat owners. To prepare a list of experts for invitation, each author proposed individuals notable for their expertise in rodent/small mammal care or research (e.g., who we had observed presenting on relevant topics at conferences/workshops or had published relevant research articles) to the lead author. We also conducted searches on Google Scholar to identify individuals who had authored rat welfare or pet rat care research articles and on Google for contributors to blogs/websites providing information about rat health and care. We continued until at least 20 individuals, with more than four individuals in each expert group, were identified (with the assumption that at least 50% would agree to participate). All invited experts met our criteria for expertise (Table 1), in addition to our criteria that no more than two persons were invited from the same place of work and that the email address for each individual was known.

**TABLE 1:** Criteria for expertise across the three groups of experts

	Expert groups		
	Animal welfare scientists	Veterinarians/veterinary nurses	Rat owners
Criteria	Contribution as first or senior author in more than three articles on rat welfare	Relevant specialist qualification (e.g., DZooMed [mammalian], DECZM [small mammals], CertVNES) AND/OR Contribution as first or senior author in more than one article on rodent health or welfare AND/OR Contribution to articles on rodent health or welfare for pet rat owners (e.g., https://ratguide.com/)	More than 3 years’ experience owning rats and having owned more than four rats AND Experience of rats outside pet ownership (e.g., laboratory technician/engagement with laboratory rat rehoming scheme/involved in rat rescue) OR Contribution to information on rodent health or welfare for pet rats owners (e.g., through personal blog)

In total, 23 individuals were invited via email to participate in the consultation, and 16 individuals agreed to participate. All but two invitations were sent to individuals in the UK, and the other invitations were sent to individuals in the United States.

### Data collection

**FIGURE 1:**
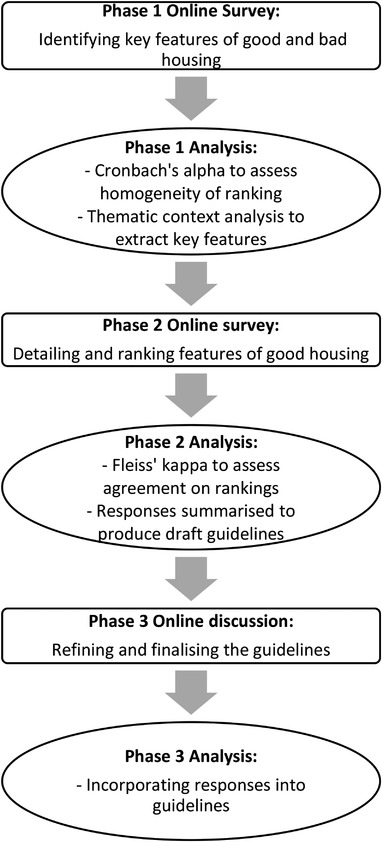
Flowchart detailing the process of developing guidelines

Data collection comprised three phases. Phases 1 and 2 involved completion of an online survey, and phase 3 involved an asynchronous online discussion (Figure 1).

### Phase 1: Identifying key features of good and bad housing

Survey 1, which was created in Google Forms, was sent to experts on 28 April 2021 and conducted for a 3‐week period. The survey was drafted by one author and refined after having been piloted and reviewed by the other authors. The aim of the first phase of the consultation was to identify key features that were present in good or bad housing so that these features could be further investigated in subsequent phases. To achieve this, we provided participants with 12 photographs of housing for pet rats that we considered to reflect the full spectrum of existing housing—ranging from cages that were likely to lead to very poor welfare to cages that were likely to provide very good welfare. These photos were obtained via two methods: (a) through an earlier survey^7^ in which rat owners were invited to send us photos of their pet rat housing and provided consent for these photos to be used to inform pet rat guidelines; (b) through an online search of images of small animal housing that were in the public domain or were licenced for sharing. Participants were asked to think about the suitability of each cage within the well‐established framework of the five freedoms.^18^ They were asked to rank each cage (on a Likert scale from 1—not at all to 7—very much) in terms of the extent to which the cage provided freedom from discomfort, freedom from pain and injury, freedom to express natural behaviours, and freedom from fear and distress (i.e., four of the five freedoms considered to be relevant to housing), in addition to ranking its overall suitability for pet rats. We also asked for a full description of the factors that led to the rating given.

To analyse these data, we first assessed the homogeneity of the ratings, as a measure of consensus, both for each cage separately and all cages jointly across the five questions. This was done using Cronbach's alpha, calculated using the Itm package in R.^37^ We then analysed the experts’ descriptions of the factors that led to the rating using thematic content analysis. To do this, we developed code in R to count the frequency of all words used in these descriptions and then manually removed all words that were not relevant to rat housing (e.g., ‘and’, ‘the’, ‘think’). We then grouped these most frequent words into themes, which formed our features for further investigation based on similarity (e.g., space: ‘space’, ‘size’, ‘small’, ‘high’).

### Phase 2: Detailing and ranking features of good housing

Survey 2 (see Table 2), which was created in Microsoft Forms, was sent to experts on 21 June 2021 and conducted for a 3‐week period. The survey was drafted by one author and refined after having been piloted and reviewed by the other authors. The aim of the second phase of the consultation was to rank the importance of the features identified in phase 1 as well as to examine how these features could lead to good or poor welfare (e.g., what is necessary for good welfare, and what is likely to lead to poor welfare?). The specific features investigated were injury prevention, digging opportunities, vertical space, horizontal space, a complex environment, food bowls/feeding areas, suitable bedding substrates, suitable nesting substrates, good ventilation, suitable materials for cage construction, opportunities to gnaw, suitable refuge area(s), opportunities to exercise, multiple levels/areas, and multiple water bottles/bowls. Participants ranked the importance of the features and were also asked questions designed to aid in the formulation of guidelines related to these features. Due to the structure of the survey platform, the ranking questions were presented in two blocks in which some questions were repeated to allow analysis of ranking across both blocks (see bottom of Table 2).

**TABLE 2:** The questions asked in survey 2, including their section and order

Question number	Section	Question
1	Good housing	How much vertical space should a cage provide?
2		How much horizontal space should a cage provide?
3		What sort of cage would provide the best ventilation?
4		What sort of bedding substrate(s) (i.e., to cover the floor of the cage) should ideally be used in the rats’ cage?
5		What sort of nesting substrate(s) (i.e., for rats to sleep on/build a nest with) should ideally be used in the rats’ cage?
6		What would be an ideal refuge?
7		What would be the best way to allow rats to climb within in cage?
8		What would be the best material(s) for the cage construction, including the flooring?
9		What is the best way to increase cage complexity?
10		What is the best way to provide rats with opportunities to exercise within the cage?
11		What would be an ideal material for gnawing?
12	Poor housing	What is the minimum vertical space a cage should provide to avoid poor welfare?
13		What is the minimum horizontal space a cage should provide to avoid poor welfare?
14		What sort of cage would provide the worst ventilation?
15		What would be the worst bedding substrate(s) to use in the cage?
16		What would be the worst nesting substrate(s) to use in the cage?
17		What cage features could potentially lead to injury?
18		What would be the worst material for the cage construction, including the flooring?
19		What cage features could potentially lead to aggression?
20	Ranking	Please rank the follow aspects of housing in terms of their importance for good rat welfare:
		A complex environment
		Good ventilation
		Horizontal space
		Injury prevention
		Multiple food bowls feeding areas
		Opportunities to gnaw
		Suitable bedding substrates
		Suitable materials for cage construction
		Suitable nesting substrates
		Vertical space
21	Ranking	Please rank the follow aspects of housing in terms of their importance for good rat welfare:
		Digging opportunities
		Horizontal space
		Injury prevention
		Multiple levels areas
		Multiple water bottles bowls
		Opportunities to exercise
		Suitable bedding substrates
		Suitable materials for cage construction
		Suitable refuge areas
		Vertical space

To assess agreement between experts about the rankings, we calculated Fleiss' kappa for each feature on each ranking question and assessed whether this was significantly different from zero using the irr package in R.^38^

Draft guidelines were developed to summarise all responses to this survey. To achieve this, the responses to the free‐text questions were first summarised according to the number of experts providing identical or highly similar (e.g., ‘split flooring’ and ‘multiple levels’; ‘ropes’ and ‘branches’) recommendations or comments about specific aspects of rat housing (e.g., ‘minimum cage height of 100 cm’; ‘dusty substrates should not be used’) for all unique recommendations and comments extracted from the given responses. The draft guidelines then comprised different sections where each section was based on each of the key features of good housing identified in survey 1, except for ‘a complex environment’ and ‘multiple levels/areas’, which were merged into one section because ‘multiple levels/areas’ was one of the most common recommendations to ensure ‘a complex environment’. Specific recommendations within each section were then populated using the recommendations/comments extracted from the survey responses. All recommendations/comments were included in the guidelines except (a) where there were conflicting recommendations (e.g., ‘minimum cage height of 100 cm’ vs. ‘minimum cage height of 120 cm’) in which case only the most popular recommendation was included; (b) where the recommendations were suggesting a specific cage material or nesting/bedding substrate that was not suitable, as we only included examples of suitable cage materials and nesting/bedding substrates; or (c) where there were a large number of examples of suitable enrichment items, cage materials, or nesting/bedding substrates, as we only included the few most popular examples to avoid unnecessarily long lists within the guidelines. No additional recommendations or comments were added. A detailed breakdown of how the guidelines were generated is provided in Supporting Information.

### Phase 3: Online discussion

Based on the results of phases 1 and 2, guidelines for pet rat housing were drafted. The aim of phase 3 was to allow the experts an opportunity to comment on these draft guidelines so that they could be refined and finalised. The online discussion, which took place using the Microsoft Teams chat function, was performed from 12 October 2021 for 25 days. Participants were provided with summary statistics of the results of the ranking questions of survey 2 (mean and standard deviation) as well as the drafted guidelines. They were asked to comment on the extent to which they agreed/disagreed with the results of the rankings and contents of the guidelines, whether they recommended any changes and whether they thought anything else had been missed. The asynchronous format allowed greater flexibility and anonymity (considered advantageous in Delphi surveys so that participants did not feel constrained in expressing their own views).^36^ All comments were then incorporated into the guidelines, leading to a refined set of guidelines.

## RESULTS

### Survey 1

Thirteen experts responded to the first survey. Of these respondents, six worked in a small animal practice (five as a qualified veterinary surgeon and one as a veterinary nurse; one of the veterinary surgeons also owned a pet rat), six had previously or currently worked in an animal research facility or veterinary department at a university (one was a qualified veterinary surgeon, one owned a pet rat, and the others were neither veterinary surgeons, veterinary nurses, nor pet rat owners), and one had never worked in academia or a small animal practice but was an experienced rat owner.

To recap, in this survey, experts were asked to rate photographs of 12 rat cages (1—not at all to 7—very much) according to the extent to which the cage provided freedom from discomfort, freedom from pain and injury, freedom to express natural behaviours, and freedom from fear and distress (i.e., four of the five freedoms considered to be relevant to housing), in addition to ranking its overall suitability for pet rats. Our analysis indicates that agreement for these ratings across participants was acceptable (defining acceptability as alpha >0.7^39^) for most of the photographs of rat cages (Table 3). This suggests that there was overall agreement about what good and poor rat housing looks like. It is unclear why agreement for cage 6 was lower.

**TABLE 3:** Cronbach's alpha and confidence interval (CI) for the expert ratings of the suitability (across five questions) of each cage and all cages jointly

Cage	Alpha	Lower CI	Upper CI
1	0.907	0.768	0.954
2	0.893	0.710	0.961
3	0.916	0.628	0.964
4	0.917	0.792	0.968
5	0.925	0.834	0.967
6	0.662	0.178	0.859
7	0.964	0.859	0.986
8	0.929	0.822	0.974
9	0.930	0.828	0.964
10	0.980	0.907	0.992
11	0.940	0.595	0.981
12	0.950	0.872	0.983
All	0.965	0.934	0.978

**FIGURE 2:**
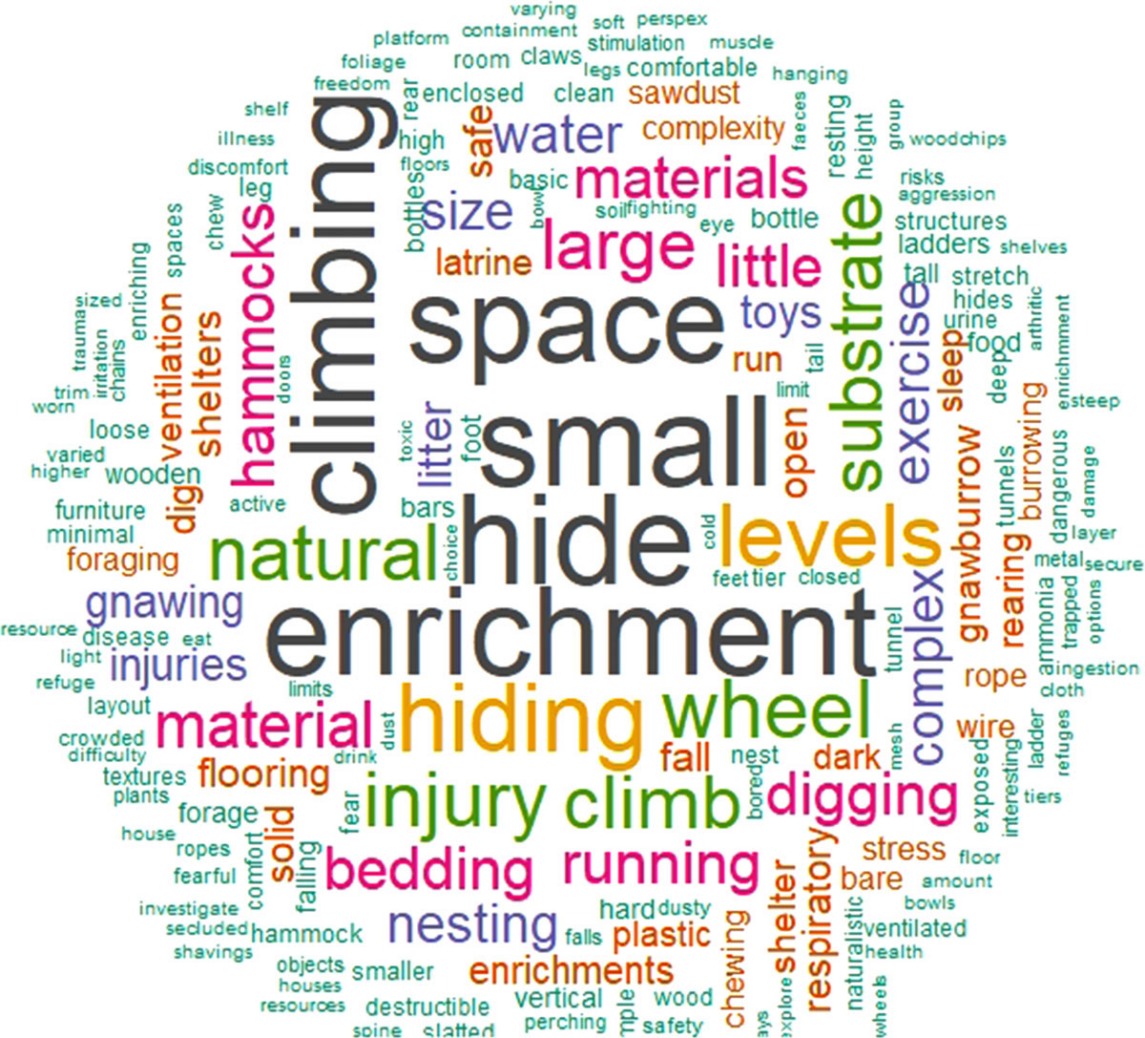
Word cloud for the description of factors underlying decisions about ratings; the size of the word represents its frequency (created using the R ‘wordcloud’ package)

In addition to the ratings, experts were asked to provide a full description of the factors that led to the rating given. Analysis of these descriptions revealed that the size of the cage, areas to hide and general enrichment levels were most commonly mentioned as factors leading to a specific rating (Figure 2).

### Survey 2

All experts who participated in survey 1 also participated in survey 2.

The experts provided a broad range of suggestions (Table 4) in response to questions about what good or unsuitable housing would entail relating to each of the key features of good/poor housing identified in survey 1 (questions 1–11 and 12–19, Table 2).

**TABLE 4:** Summary of the recommendations/comments extracted from the responses to each open‐text question in survey 2

Factor and questions	Themes (extract theme—number of respondents)
*Suitable refuge areas*: What would be an ideal refuge?	Hammock—7
	Hides/boxes—7
	Cardboard—5
	Fabric/soft—5
	Multiple/variety—5
	Plastic—5
	Tunnels/tubes—5
	Wooden—4
	Dark/enclosed—3
	Washable/disposable—3
	Large enough for more than one rat/space to turn around/bring nesting material inside—2
	Single exit—2
	Breathable—1
	Burrow like—1
	Multiple exits—1
	Solid—1
*Vertical space*: How much vertical space should a cage provide?/What is the minimum vertical space a cage should provide to avoid poor welfare?	Space for rearing/stretching is important—12
	At least three body lengths (e.g., 90–120 cm)—9
	Split flooring/multiple levels are important—9
	Sufficient to allow climbing—8
	Bigger is better—4
	At least one body length (e.g., between 30 cm and 60 cm)—3
	At least two body lengths (e.g., between 60 cm and 90 cm)—2
	Fall breakers are important—2
	Natural behaviours—2
*A complex environment*: What is the best way to increase cage complexity?	Multiple levels/tiers/floors—8
	Multiple obstacles/toys/objects/enrichment items—6
	Multiple food and/or water stations/foraging opportunities/puzzle feeders—4
	Multiple refuges—4
	Opportunities to dig/burrow—4
	Opportunities to climb—4
	Multiple substrates—3
	Sufficient space—3
	Tunnels—2
	Opportunities to nest—1
*Opportunities to exercise*: What is the best way to provide rats with opportunities to exercise within the cage?/What would be the best way to allow rats to climb within in cage?	Ropes/branches/ladders—13
	Multiple levels (e.g., including platforms and hammocks)—11
	Running wheel—9
	Opportunities to climb—8
	Foraging activities—5
	Sufficient space—4
	Cage wire allows climbing—3
	Fall breakers are important—2
	Nesting opportunities—1
	Digging opportunities—1
*Injury prevention*: What cage features could potentially lead to injury?/What cage features could potentially lead to aggression?	Sufficient space reduces injury/aggression—9
	Sufficient refuge areas reduce injury/aggression—7
	Sufficient resources/feeding areas/drinking areas reduce injury/aggression—6
	Use of fall breakers reduce injury—6
	Sufficient enrichment items reduce injury/aggression—5
	Sharp edges (e.g., from chewed plastic) need to be avoided—5
	Unsafe wheels (e.g., open wired/slatted or too small) should not be used—4
	Unsuitable cage materials need to be avoided (e.g., wire/slatted flooring that causes pododermatitis/traps feet)—4
	Materials that could cause injury/death through ingestion should be avoided (e.g., lead/zinc/rubber/plastic)—3
	Materials that could cause constriction injuries (e.g., chain of metal/bedding) should be avoided—2
	Sufficiently regular cleaning reduces injury—1
	Sufficient ventilation reduced injury—1
	Unsafe substrates (e.g., causes respiratory disease) should be avoided—1
*Horizontal space*: How much horizontal space should a cage provide?/What is the minimum horizontal space a cage should provide to avoid poor welfare?	At least three body lengths (e.g., 90–120 cm)—11
	Sufficient to allow scampering/running/exercise—9
	As big as possible—5
	At least two body lengths (e.g., 60–90)—5
	Sufficient to allow exploration/foraging—3
	Sufficient to allow social activities (e.g., rough and tumble play/communal sleeping)—3
	Sufficient to allow different zones—1
*Good ventilation*: What sort of cage would provide the best ventilation?/What sort of cage would provide the worst ventilation?	Not fully enclosed (e.g., avoiding glass/plastic tanks or vivariums)—15
	Open bars (e.g., wire/mesh)—12
	Location in room important/avoid drafts—5
*Suitable nesting substrates*: What sort of nesting substrate(s) (i.e., for rats to sleep on/build a nest with) should ideally be used in the rats cage?/What would be the worst nesting substrate(s) to use in the cage?	Paper/tissue/paper wool—10
	Dust free—9
	Substrates that would not cause injury through constriction/catching claws/ingestion (e.g., cotton balls/long strands)—6
	Shredded materials (including material that rats can shred themselves)—4
	Fleece (good)—4
	Cotton wool (bad)—3
	Soft material—3
	Wood (bad)—3
	Not noisy—2
	Long strips—2
	Hay/straw (bad)—2
	Hay/straw (good)—2
	Cat litter (bad)—1
	Not absorbent—1
	Dried leaves—1
	Soil—1
	Hammock—1
	Easy to clean—1
*Suitable bedding substrates*: What sort of bedding substrate(s) (i.e., to cover the floor of the cage) should ideally be used in the rats cage?/What would be the worst bedding substrate(s) to use in the cage?	Dust free/does not cause respiratory issues—17
	Paper based (good)—11
	Absorbent—10
	Wood shavings/sawdust (bad)—9
	Digging/burrowing—5
	Wood shavings/sawdust (good)—5
	Coco‐soil/soil—4
	Odour free—4
	Allows foraging—3
	Fleece (bad)—3
	Does not cause constriction injuries—2
	Fabric (good)—2
	More than one/variety—2
	Paper based (bad)—2
	Regularly changed—2
	Naturalistic—1
	Hemp—1
	Easy to clean—1
	Avoid rough materials—1
	Avoid solid materials (e.g., bare plastic)—1
	Avoid toxic materials—1
	Noisy—1
	Hay (bad)—1
*Suitable materials for cage construction*: What would be the best material(s) for the cage construction, including the flooring?/What would be the worst material for the cage construction, including the flooring?	Metal (good)—12
	Plastic (good)—10
	Avoid wire/slatted flooring—8
	Wood (bad)—7
	Easy to clean/hygienic—6
	Plastic (bad)—6
	Bars for walls (good)—5
	Chewable materials should be avoided—5
	Glass (bad)—3
	Nontoxic—3
	Wood (good)—2
	Materials that cause injuries (e.g., pododermatitis) should be avoided—2
	Metal (bad)—2
	Ventilation (e.g., not solid sides)—2
	Avoid cold materials for flooring—1
	Allows climbing—1
	Provides shelter from overhead lighting—1
*Opportunities to gnaw*: What would be an ideal material for gnawing?	Wood—13
	Cardboard—2
	Avoid rubber—1
	Animal bones—1
	Hard‐shelled nuts—1
	Dog biscuit—1
	Rope—1

When experts were then asked to rank the key features of good/bad housing identified in survey 1 (listed in questions 20 and 21 of Table 2), there was wide variation in the extent to which each feature was considered important—with at least one expert ranking each feature within the top three, and at least one expert ranking each feature within the bottom three (Table 5). Moreover, there was poor agreement for all features according to Fleiss’ kappa (kappa < 0.4—poor; 0.4 < kappa < 0.75—fair; kappa > 0.75—excellent^40^) with the majority of values not being significantly different from zero (except in the case of digging opportunities and vertical space; Table 6). This suggests a lack of consensus among the experts as to which feature is most important or that a ranking methodology was inappropriate to evaluate the importance of these features.

**TABLE 5:** The mean rank within each ranking question, mean across both questions (20 and 21 of Table 2), and minimum and maximum ranking for the different factors related to rat housing

Feature	Q1 mean rank	Q2 mean rank	Mean rank	Min	Max
Suitable refuge areas		3.077	3.077	1	7
Vertical space	3.077	3.692	3.385	1	8
Multiple levels/areas		3.846	3.846	1	7
A complex environment	4.538		4.538	1	10
Opportunities to exercise		4.769	4.769	2	9
Injury prevention	4.538	5.077	4.808	1	10
Horizontal space	4.462	5.308	4.885	1	10
Good ventilation	5.231		5.231	1	10
Suitable nesting substrates	6.154		6.154	3	10
Suitable bedding substrates	6.231	6.923	6.577	1	10
Multiple water bottles/bowls		7.308	7.308	1	10
Digging opportunities		7.385	7.385	1	10
Suitable materials for cage construction	6.231	7.615	6.923	2	10
Multiple food bowls/feeding areas	7.077		7.077	2	10
Opportunities to gnaw	7.462		7.462	3	10

**TABLE 6:** Statistical analysis of the rankings for each factor for both ranking questions using Fleiss’ kappa

Question	Feature	Kappa	*z*‐Value	*p*‐Value
Q1	A complex environment	0.017	0.477	0.633
Q1	Good ventilation	−0.040	−1.114	0.265
Q1	Horizontal space	0.017	0.477	0.633
Q1	Injury prevention	−0.026	−0.716	0.474
Q1	Multiple food bowls feeding areas	0.003	0.080	0.937
Q1	Opportunities to gnaw	−0.011	−0.318	0.750
Q1	Suitable bedding substrates	0.003	0.080	0.937
Q1	Suitable materials for cage construction	−0.054	−1.512	0.131
Q1	Suitable nesting substrates	−0.026	−0.716	0.474
Q1	Vertical space	0.060	1.671	0.095
**Q2**	**Digging opportunities**	**0.074**	**2.069**	**0.039**
Q2	Horizontal space	0.003	0.08	0.937
Q2	Injury prevention	0.031	0.875	0.381
Q2	Multiple levels/areas	0.017	0.477	0.633
Q2	Multiple water bottles bowls	0.06	1.671	0.095
Q2	Opportunities to exercise	−0.026	−0.716	0.474
Q2	Suitable bedding substrates	−0.011	−0.318	0.75
Q2	Suitable materials for cage construction	0.046	1.273	0.203
Q2	Suitable refuge areas	0.06	1.671	0.095
**Q2**	**Vertical space**	**0.074**	**2.069**	**0.039**

Bold values denote statistical significance (*p* < 0.05).

### Online discussion

A total of eight experts contributed to the online discussion: one veterinary nurse working in a small animal practice, one veterinary surgeon working in academia, one veterinary surgeon working in a small animal practice, four working in academia who were neither veterinary nurses nor veterinary surgeons, and one experienced rat owner who was not a veterinary nurse, veterinary surgeon or working in academia. In the online discussion, experts discussed their views on the draft guidelines. There were a number of points made about the contents of the draft guidelines that other experts agreed with: suggestions to scatter‐feed rather than provide food in bowls, to provide rats with time outside the cage, to add crinkle‐cut paper to the list of potential nesting materials, and to modify cages for infirm or aged animals. There were also some suggestions for clarity and detail in the guidelines, which were provision of more detailed information about providing digging opportunities and clarification on the bedding material examples, both with suggestions on how to achieve this. These points raised were then included in the draft guidelines that were developed to prepare the finalised guidelines (Table 7, also see Supporting Information for details on specifically how and where these points were addressed). Several participants expressed concerns about the mean ranking of the features of good/bad housing: two thought that opportunities to gnaw should be ranked more highly, two thought that complex cages should be ranked more highly, one thought that opportunities to dig should be ranked more highly and one thought that horizontal space should be prioritised over vertical height. Due to these concerns and the poor agreement about the rankings in the responses to survey 2, the final guidelines did not include any information about which features might be of highest importance.

**TABLE 7:** Finalised guidelines for the housing of pet rats

Necessary and important components of rat housing	How these can be achieved (assuming two to four rats per cage)
Consideration must be given to the age and agility of the rat	The advice here may need to be adjusted for rats with poorer balance or agility, or who are infirm. For example, ramps rather than climbing ropes may be most appropriate for older and infirm rats.
Rats must be provided with a complex environment that includes multiple tiers/levels	Rats should be provided with a cage that has a minimum of two tiers. But ideally, rats should be provided with multiple tiers, distinct areas within the cage, and multiple enrichment items. Tubes could be used to provide burrow‐like areas, as well as provide some degree of refuge. Hammocks can be used to provide additional levels. Complexity can also be increased by periodically rearranging enrichment items, except for refuge areas, which should not be moved, and by adding novel items to the cage.
Rats must be provided with digging opportunities	This could be provided via suitable bedding material. In addition to suitable bedding material, digging boxes should be included within the cage. For example, a storage box with a rat‐sized hole in the lid and side could be filled with coco‐soil or crinkle‐cut paper to a depth of at least one body length. Smaller containers could also be buried within this substrate to mimic chambers within a rat burrow.
Rats must be provided with multiple food bowls/feeding areas	Multiple feeding areas are recommended to avoid aggression. Foraging toys or scattering food through the cage may be preferable to bowls. For example, hiding food within a cardboard egg box.
Rats must be provided with multiple water bottles/bowls	Multiple water bottles/bowls are recommended to avoid aggression.
Rats must be provided with opportunities to exercise	A safe (solid flooring) and large (>16 in.) running wheel and climbing opportunities (e.g., via ropes, platforms, branches, ladders) will provide opportunities to exercise. Rats should be provided with a minimum of 1–2 hours per day outside the cage in a safe space, that includes refuge areas, to allow exploration. Foraging toys and scatter feeding within the cage may also incentivise exercise.
Rats must be provided with refuge areas	A refuge should, as a minimum, provide a dark and enclosed area that is large enough to fit multiple rats. Multiple and varied refuge areas are recommended. Refuges could vary in terms of the number of exits, material, and size. Washable or disposable refuges are preferable. Examples include carboard boxes (which also allow rats to chew their own exits), enclosed fabric hammocks and solid plastic hides.
Rats must be provided with sufficient horizontal space	Anything below 90–120 cm in width (at least three body lengths) is not suitable, and much larger cages are advised. Rats should be provided with as much horizontal space as possible, to provide them with an opportunity to scamper/run, engage in social activities and explore.
Rats must be provided with sufficient vertical space	Anything below 90–120 cm in height (at least three body lengths) is not suitable, and much larger cages are advised. Rats should be provided with as much vertical space as possible, and provided with multiple tiers within a cage that allow them opportunities to engage in natural behaviours such as climbing and rearing. Items that are soft and wide, such as hammocks, should be provided to act as fall breakers.
Rats must be provided with suitable bedding substrates	Bedding substrates that are dust free, absorbent, soft, non‐scented, non‐toxic and do not cause injuries (e.g., via constriction) are ideal. Bedding should be composed of particles (i.e., not solid) that covers the entire cage floor to allow rats an opportunity to engage in natural behaviours like digging, burrowing and foraging for food left on the cage floor, and more than one type of bedding is ideal to provide variety. Examples of ideal bedding are as follows: compressed paper, coco‐soil and dust‐free aspen chips. Bedding must be cleaned or replaced sufficiently regularly to avoid build‐up of ammonia and faeces.
Rats must be provided with suitable nesting substrates	Dusty substrates and those which could cause injuries (i.e., via constriction or ingestion) are not advised. Ideal nesting materials should be soft, absorbent and be composed of long strips (e.g., shredded materials). Examples include paper wool or crinkle‐cut paper. Non‐soiled nesting material should be kept intact and not be replaced during cage changes.
Suitable materials for cage construction must be used	Materials that provide poor ventilation or are difficult to clean are not advised (e.g., vivariums/wooden floors are not advisable). Cages with plastic solid flooring and uncoated metal bars are ideal to avoid injuries, for ease of cleaning and for ventilation.
The cage must be designed to reduce the risk of injury	To avoid injuries, wire flooring should be avoided, soft and wide fall breakers (e.g., hammocks) should be provided, the cage should be monitored for sharp edges from chewed plastic and any materials that could cause constriction injuries or can catch claws or teeth (e.g., loose weave long fibre fabrics/fluffy bedding/metal chains) should be avoided. Likewise, materials that could pose problems if ingested should be avoided, including rubber objects. Metal bars on the cage must not be coated with zinc or galvanised to avoid metal toxicosis. Wooden items provide gnawing opportunities to avoid overgrown teeth.
The location of the cage must be suitable	The cage must be located in an area that is not draughty or too cold, that does not get too hot, is not too bright (e.g., not directly below a light), and that is not near any sources of loud noise, including ultrasonic noises (e.g., next to a television, music system, electronic equipment), or other stressors (e.g., predator species like cats).

## DISCUSSION

Providing owners with good and accurate information about the husbandry of their pets is vitally important for companion animal welfare. However, this can be challenging when evidence about good husbandry is poor or lacking, as in the case of pet rats. To obviate this issue, we aimed to develop guidelines for pet rat housing based on expert opinion by consulting experienced pet rat owners, veterinarians, veterinary nurses and animal welfare researchers (including those focused on laboratory rodent welfare). The development of these guidelines involved a set of surveys to determine the core features that were important to good housing for pet rats.

We achieved the aim of developing guidelines for pet rat housing. These guidelines comprise 14 key factors that were considered important and necessary for good housing. Namely, consideration must be given to the age and agility of the rat, rats must be provided with a complex environment that includes multiple tiers/levels, rats must be provided with digging opportunities, rats must be provided with multiple food bowls/feeding areas, rats must be provided with multiple water bottles/bowls, rats must be provided with opportunities to exercise, rats must be provided with refuge areas, rats must be provided with sufficient horizontal space, rats must be provided with sufficient vertical space, rats must be provided with suitable bedding substrates, rats must be provided with suitable nesting substrates, suitable materials for cage construction must be used, the cage must be designed to reduce the risk of injury, and the location of the cage must be suitable. The guidelines also provide details about how each of these can be achieved. Although these guidelines were developed through expert consultation, they are open to evolve as more evidence and information about pet rats comes to light and should not be considered definitive. Importantly, we recommend that these guidelines be regularly revisited to ensure that they remain relevant and up to date. In the meantime, the more immediate challenge will be the dissemination of this information to pet rat owners.

Although the aim of this study was to develop guidelines for pet rats, these guidelines may also be useful in the development of revised guidelines and codes of conduct for laboratory rodents. For example, the minimum conditions outlined in the United Kingdom Home Office Code of Practice allow much more restrictive housing than the conditions outlined in the guidelines we have developed, such as a minimum height of cage of 20 cm and a minimum floor area of 400 cm^2^ (per animal)—such that a cage of width and length 40 cm would be acceptable for four rats (our guidelines advise a height and width of 120 cm) and no mention of multiple levels/compartments or opportunities to exercise (which are included in our guidelines).^41^ Inevitably, there may be more practical considerations for laboratory rats compared to pet rats, such as fitting a large number of rodents in a smaller space and ensuring that rats are easy to observe and that checks can be rapidly conducted. However, it is nevertheless important to ask the question of whether and in what ways guidelines for the welfare of the same species should vary according to the purposes for which they are being kept. Indeed, this issue is just one part of the continuing progression of animal welfare regulation; for example, the need for laboratory rats to be able to rear fully in their cages and the use of playpens for enrichment have received growing attention.^8^, ^9^, ^12^, ^42^, ^43^

During the development of these guidelines, there were some areas of conflict. For example, there was much disagreement about how to rank the different aspects of rat housing. This may reflect that many experts considered all the features listed to be vitally important for good rat housing, leading to difficulties when ranking them. It may also reflect that the different backgrounds of the experts may have influenced what they considered to be most important. For example, those who more frequently observe rat injuries may have given injury prevention a higher ranking, while those who more commonly observe rats in their home cage may rate opportunities to engage in particular behaviours (such as digging or nesting) more highly (i.e., because they frequently observe these behaviours).

Additionally, while experts were able to reach consensus on cage dimensions that were too small, there was no consensus for cage dimensions that should be recommended beyond a suggestion that cages should be as big as possible. This could potentially reflect that there is too little information or evidence on which to base these recommendations, especially given that an online search for commercially available pet rat cages revealed that all rat cages (and many cages for larger pets such as ferrets and rabbits) do not or only just meet the minimum recommended cage size outlined in our guidelines. Importantly, the home range for wild brown rats has been found to be a minimum of ∼30 m in diameter.^44^ This suggests that it would be impossible or very challenging to allow pet rats sufficient space so that they are not restricted in comparison to their wild counterparts. Further research would be valuable to assess the amount of space rats require to ensure good welfare.

Additionally, although we attempted to include experts across different backgrounds, it is important to note that there are additional groups who were not represented in the consultation but who may have had valuable input (e.g., ecologists or laboratory technicians). Likewise, although the number of experts recruited was within recommendations,^24^, ^36^ the guidelines development may, in the future, benefit from a larger number of experts with a broader range of experience. Accordingly, it will be important and necessary to conduct larger scale studies to further examine and build upon our guidelines with a larger and more comprehensive pool of experts.

To conclude, through expert consultation, we have developed a set of guidelines for pet rat housing. These guidelines cover a broad range of features within pet rat housing, including injury prevention, details of suitable refuges and substrates and suitable cage sizing. We hope that these guidelines will not only be useful for pet rat owners but may also be useful in the future design of housing for laboratory rodents and that they will provide a useful starting point for future guidelines for rat housing as more evidence about appropriate rat husbandry emerges.

## CONFLICT OF INTEREST

The authors declare they have no conflict of interest.

## ETHICS STATEMENT

This study received ethical approval from the Faculty of Health Sciences Ethics Committee at the University of Bristol (reference number: 103082). All experts provided informed consent for their participation in the survey.

## AUTHOR CONTRIBUTIONS

*Conceptualisation* *, methodology, and writing (reviewing and editing)*: all authors. *Data collection and analysis, and writing (first draft)*: Vikki Neville.

## Supporting information

Supporting Information

## Data Availability

The raw survey data that support the findings of this study are available from the corresponding author upon reasonable request.
