# Supplementary material for: Developing guidelines for pet rat housing through expert consultation
Source: Vet Rec. 2022 Jul 16;192(3):e1839. doi: 10.1002/vetr.1839 (PMC10084028; doi:10.1002/vetr.1839)
Supplement: Supplementary file 1 — Supporting Information [file VETR-192-no-s001.docx]

| **Necessary and important components of rat housing** | **Source of ‘Necessary and important components of rat housing’** | **How these can be achieved (assuming 2-4 rats per cage)** | **Source of ‘How these can be achieved (assuming 2-4 rats per cage)’** |
| --- | --- | --- | --- |
| Consideration must be given to the age and agility of the rat. | **Online discussion:**  “Should there also be a comment to cover that the enclosure should be monitored and modified according to the agility/age of the rat?” | The advice here may need to be adjusted for rats with poorer balance or agility, or who are infirm. For example, ramps rather than climbing ropes may be most appropriate for older and infirm rats. | **Online discussion:**  “.. as they become older climbing ropes/ladders should be replaced by ramps etc?” |
| Rats must be provided with a complex environment that includes multiple tiers/levels | **Important features identified in survey 1 & 2:**  A complex environment  Multiple levels/areas | Rats should be provided with a cage that has a minimum of two tiers. But ideally, rats should be provided with multiple tiers, distinct areas within the cage, and multiple enrichment items. Tubes could be used to provide burrow-like areas, as well as provide some degree of refuge. Hammocks can be used to provide additional levels. Complexity can also be increased by periodically re-arranging enrichment items, except refuge areas which should not be moved, and by adding novel items to the cage. | **Survey 2, Q9** - **What is the best way to increase cage complexity?:**  Multiple levels/tiers/floors – 8 (*Included*)  Multiple obstacles/toys/objects/enrichment items – 6 (*Included*)  Multiple food and/or water stations/foraging opportunities/puzzle feeders – 4 (*Included under ‘Rats must be provided with multiple food bowls/feeding areas’*)  Multiple refuges – 4 (*Included under ‘Rats must be provided with refuge areas’*)  Opportunities to dig/burrow – 4 (*Included under ‘Rats must be provided with digging opportunities’*)  Opportunities to climb – 4 (*Included under ‘Rats must be provided with opportunities to exercise’)*  Multiple substrates – 3 (*Included under ‘Rats must be provided with suitable bedding substrates’*)  Sufficient space – 3 (*Included under ‘Rats must be provided with sufficient horizontal space’ and ‘Rats must be provided with sufficient vertical space’*)  Tunnels – 2 (*Included)*  Opportunities to nest – 1 (*Included under ‘Rats must be provided with suitable nesting substrates*)  **Online discussion:** “*Multiple layers/ complex environment - can we include hammocks as an example?”*  *“complexity can be increased through novelty or periodically rearranging furniture (bearing in mind that refuges should usually remain stable because they provide security)?”* |
| Rats must be provided with digging opportunities | **Important features identified in survey 1 & 2:**  Digging opportunities | This could be provided via suitable bedding material. In addition to suitable bedding material, digging boxes should be included within the cage. For example, a storage box with a rat-sized hole in the lid and side could be filled with coco-soil or crinkle-cut paper to a depth of at least 1 body length. Smaller containers could also be buried within this substrate to mimic chambers within a rat burrow. | **Survey 2, Q4 & Q15 - What sort of bedding substrate(s) should ideally be used in the rats cage? & What would be the worst bedding substrate(s) to use in the cage?:**  “Digging/burrowing – 5”  **Online discussion:**  *“both practical and very nice is to include a closed tupperware with coconut fiber substrate, crinkle cut paper, actual dirt, or paper wool that is filled to at least 1 body length deep to allow for digging. This container can also include smaller containers within it to mimic burrows”* |
| Rats must be provided with multiple food bowls/feeding areas | **Important features identified in survey 1 & 2:**  Multiple food bowls feeding areas | Multiple feeding areas are recommended to avoid aggression. Foraging toys or scattering food through the cage may be preferable to bowls. For example, hiding food within a cardboard egg box. | **Survey 2, Q17 - What cage features could potentially lead to injury?:**  Sufficient resources/feeding areas/drinking areas - 6  **Survey 2, Q9** - **What is the best way to increase cage complexity?:**  Multiple food and/or water stations/foraging opportunities/puzzle feeders – 4  **Online discussion:**  “recommend for people to not feed out of a bowl and instead use foraging toys. For example, using an old cardboard egg carton and nesting material to hide feed in so the rats need to forage for it”  “bowl feeding is boring and the rats can get some much from working for the food, either foraging or removing from boxes etc”  “worth mentioning scatter feeding as an incentive for rats to move around”  “prevent aggression, but also encourages natural foraging behaviour and exercise |
| Rats must be provided with multiple water bottles/bowls | **Important features identified in survey 1 & 2:**  Multiple water bottles bowls | Multiple water bottles/bowls are recommended to avoid aggression | **Survey 2, Q17 - What cage features could potentially lead to injury?:**  Sufficient resources/feeding areas/drinking areas - 6 |
| Rats must be provided with opportunities to exercise | **Important features identified in survey 1 & 2:**  Opportunities to exercise | A safe (solid flooring) and large (>16 inches) running wheel, and climbing opportunities (e.g. via ropes, platforms, branches, ladders) will provide opportunities to exercise. Rats should be provided with a minimum of 1-2 hours per day outside the cage in a safe space, that includes refuge areas, to allow exploration. Foraging toys and scatter feeding within the cage may also incentivise exercise. | **Survey 2, Q7 & Q10 - What would be the best way to allow rats to climb within in cage? & What is the best way to provide rats with opportunities to exercise within the cage?:**  Ropes/branches/ladders -13 (*Included)*  Multiple levels (e.g. including platforms and hammocks) – 11 (*Included under ‘Rats must be provided with a complex environment that includes multiple tiers/levels’)*  Running wheel – 9 (*Included)*  Opportunities to climb – 8 (*Included)*  Foraging activities – 5 (*Included)*  Sufficient space – 4 (*Included under ‘Rats must be provided with sufficient horizontal space’ and ‘Rats must be provided with sufficient vertical space’*)  Cage wire allows climbing – 3 (*Not included – more popular examples given/Recommendation for wire bars included under ‘Suitable materials for cage construction must be used’)*  Fall breakers are important - 2 (*Included under ‘The cage must be designed to reduce the risk of injury’)*  Nesting opportunities – 1 (*Included under ‘Rats must be provided with suitable nesting substrates*)  Digging opportunities – 1 (*Included under ‘Rats must be provided with digging opportunities’*)  **Online discussion:**  “*should include time out of the cage for the rats (suggest minimum 1-2 hours a day?)”*  “worth mentioning scatter feeding as an incentive for rats to move around”  “mention the importance of space, including safe out-of-cage exploration if the cage itself does not provide space”  “complexity can additionally be offered through outside cage exploration of safe environments, e.g. rat proof playpens” |
| Rats must be provided with refuge areas | **Important features identified in survey 1 & 2:**  Suitable refuge areas | A refuge should, as minimum, provide a dark and enclosed area that is large enough to fit multiple rats. Multiple and varied refuge areas are recommended. Refuges could vary in terms of the number of exits, material, and size. Washable or disposable refuges are preferable. Examples include: carboard boxes (which also allow rats to chew their own exits), enclosed fabric hammocks, and solid plastic hides. | **Survey 2, Q6 - What would be an ideal refuge?**  Hammock – 7 (*Included)*  Hides/boxes – 7 (*Included)*  Cardboard – 5 (*Included)*  Fabric/soft – 5 (*Included)*  Multiple/variety – 5 (*Included)*  Plastic – 5 (*Included)*  Tunnels/tubes – 5 (*Included under ‘Rats must be provided with a complex environment that includes multiple tiers/levels’)*  Wooden – 4 (*Not included – more popular examples given*)  Dark/enclosed – 3 (*Included)*  Washable/disposable – 3 (*Included)*  Large enough for more than one rat/space to turn around/bring nesting material inside - 2 (*Included)*  Single exit – 2 (*Partially included – conflict with suggestion for multiple exists)*  Breathable – 1 (*Partially included as recommendation is ambiguous and received little support – examples given all allow adequate air supply)*  Burrow-like – 1 (*Included under ‘Rats must be provided with a complex environment that includes multiple tiers/levels’)*  Multiple exits – 1 (*Partially included – conflict with suggestion for multiple exists)*  Solid – 1 (*Included)* |
| Rats must be provided with sufficient horizontal space | **Important features identified in survey 1 & 2:**  Horizontal space | Anything below 90-120cm in width (at least three body lengths) is not suitable, and much larger cages are advised. Rats should be provided with as much horizontal space as possible, to provide them with an opportunity to scamper/run, engage in social activities, and explore. | **Survey 2, Q2 & Q13 - How much horizontal space should a cage provide? & What is the minimum horizontal space a cage should provide to avoid poor welfare?**  At least 3 body lengths (e.g. 90-120cm) – 11 (*Included)*  Sufficient to allow scampering/running/exercise – 9 (*Included)*  As big as possible – 5 (*Included)*  At least 2 body lengths (e.g. 60-90) – 5 (*Not included – conflict with recommendation of at least 3 body lengths*)  Sufficient to allow exploration/foraging – 3 (*Included)*  Sufficient to allow social activities (e.g. rough and tumble play/communal sleeping) – 3 (*Included)*  Sufficient to allow different zones – 1 (*Included under ‘Rats must be provided with a complex environment that includes multiple tiers/levels’)*  **Online discussion:**  “The minimum height and width of cages as 3 body lengths is way too small. I would be advising a larger cage if an owner had a pet rat in a cage that small”  “rats need to run as well as climb” |
| Rats must be provided with sufficient vertical space. | **Important features identified in survey 1 & 2:**  Vertical space | Anything below 90-120cm in height (at least three body lengths) is not suitable, and much larger cages are advised. Rats should be provided with as much vertical space as possible, and provided with multiple tiers within a cage that allow them opportunities to engage in natural behaviours such as climbing and rearing. Items that are soft and wide, such as hammocks, should be provided to act as fall breakers. | **Survey 2, Q1 & Q12 - How much vertical space should a cage provide? & What is the minimum vertical space a cage should provide to avoid poor welfare?**  Space for rearing/stretching is important – 12 (*Included)*  At least 3 body lengths (e.g. 90-120cm) – 9 (*Included)*  Split flooring/multiple levels are important – 9 (*Included)*  Sufficient to allow climbing – 8 (*Included)*  Bigger is better – 4 (*Included)*  At least 1 body length (e.g. >30 <60cm) -3 (*Not included – conflict with recommendation of at least 3 body lengths*)  At least 2 body lengths (e.g. 60-90) – 2 (*Not included – conflict with recommendation of at least 3 body lengths*)  Fall breakers are important – 2 (*Included)*  Natural behaviours - 2 (*Included)*  **Online discussion:**  “there is a risk of falling with very tall cages”  “the minimum height and width of cages as 3 body lengths is way too small. I would be advising a larger cage if an owner had a pet rat in a cage that small” |
| Rats must be provided with suitable bedding substrates | **Important features identified in survey 1 & 2:**  Suitable bedding substrates | Bedding substrates that are dust-free, absorbent, soft, non-scented, non-toxic, and do not cause injuries (e.g. via constriction) are ideal. Bedding should be composed of particles (i.e., not solid) that covers the entire cage floor to allow rats an opportunity to engage in natural behaviours like digging, burrowing, and foraging for food left on the cage floor, and more than one type of bedding is ideal to provide variety. Examples of ideal bedding are: compressed paper, coco-soil, and dust-free aspen chips. Bedding must be cleaned or replaced sufficiently regularly to avoid build up of ammonia and faeces. | **Survey 2, Q4 & Q15 - What sort of bedding substrate(s) (i.e. to cover the floor of the cage) should ideally be used in the rats cage? & What would be the worst bedding substrate(s) to use in the cage?**  Dust-free/doesn’t cause respiratory issues – 17 *(Included)*  Paper-based (good) – 11 *(Included)*  Absorbent – 10 *(Included)*  Wood shavings/sawdust (bad) – 9 (*Not included – only good examples provided*)  Digging/burrowing – 5 *(Included)*  Wood shavings/sawdust (good) – 5 (*Not including – conflict with advice against wood shavings/sawdust*)  Coco-soil/soil – 4 *(Included)*  Odour free – 4 *(Included)*  Allows foraging - 3 *(Included)*  Fleece (bad) – 3 (*Not included – only good examples provided*)  Doesn’t cause constriction injuries – 2 *(Included)*  Fabric (good) -2 (*Not included – more popular examples provided)*  More than one/variety- 2 *(Included)*  Paper-based (bad) – 2 (*Not included – conflict with advice that paper is good)*  Regularly changed – 2 *(Included)*  Naturalistic – 1 (*Included)*  Hemp - 1 (*Not included – more popular examples included*)  Easy to clean – 1 (*Partially included – all examples given are disposable and recommendation for regular cleaning)*  Avoid rough materials -1 *(Included)*  Avoid solid materials (e.g. bare plastic) – 1 *(Included)*  Avoid toxic materials – 1 (*Included)*  Noisy - 1 (*Partially included as recommendation is ambiguous and received little support – examples are not noisy materials)*  Hay (bad) – 1 (*Not included – only good examples provided)*  **Online discussion:**  “I know a lot of people use dust free wood shavings and don't seem to get any respiratory issues.”  “I've seen people use lavender scented or lemon scented wood shavings and have seen problems (whether resp issues solely due to this, or other underlying issues, I don't know)”  “I would also add aspen chips to this list as well as recycled paper bedding. Basically something like CareFresh, but when it says "pellets" I think harder substances.”  “I agree that pellets can refer to very hard, cylindrical pieces. Maybe 'compressed paper' bedding is better wording? I'm not sure I'd recommend Carefresh, though - it seems fine as nesting material, but is not absorbent enough as a bedding, unless you use a very large volume of it. It's quite dusty too.” |
| Rats must be provided with suitable nesting substrates | **Important features identified in survey 1 & 2:**  Suitable bedding substrates | Dusty substrates and those which could cause injuries (i.e. via constriction or ingestion) are not advised. Ideal nesting materials should be soft, absorbent, and be composed of long strips (e.g. shredded materials). Examples are: paper wool or crinkle cut paper. Non-soiled nesting material should be kept intact and not be replaced during cage changes. | **Survey 2, Q5 & Q116 - What sort of nesting substrate(s) (i.e. to cover the floor of the cage) should ideally be used in the rats cage? & What would be the worst nesting substrate(s) to use in the cage?**  Paper/tissue/paper wool – 10 *(Included)*  Dust-free – 9 *(Included)*  Substrates that would not cause injury through constriction/catching claws/ingestion (e.g. cotton balls/long strands) – 6 *(Included)*  Shredded materials (including material that rats can shred themselves) – 4 *(Included)*  Fleece (good) – 4 (*Not included – more popular examples given)*  Cotton wool (bad) – 3 (*Not included – only good examples provided)*  Soft material – 3 *(Included)*  Wood (bad) – 3 (*Not included – only good examples provided)*  Not noisy – 2 (*Partially included as recommendation is ambiguous and received little support – examples given are not noisy materials)*  Long strips – 2 *(Included)*  Hay/straw (bad) – 2 (*Not included – only good examples provided)*  Hay/straw (good) – 2 (*Not included – conflict with advice against hay)*  Cat litter (bad) – 1 (*Not included – only good examples provided)*  Not absorbent – 1 *(Included)*  Dried leaves – 1 (*Not included – more popular examples given)*  Soil – 1 (*Not included – more popular examples given)*  Hammock – 1 (*Not included – more popular examples given)*  Easy to clean – 1 (*Partially included – all examples given are disposable)*  **Online discussion:**  “I would also add crinkle cut paper to the list. My pet rats (as well as many lab rats) will use that quite well. And it's pretty cheap and practical. It might also be nice to add a recommendation to keep nesting material intact during cage changes.”  “I would agree with this addition. However, I have seen some male rats consume it but it didn't cause any compactions (in the lab). The literature says that if they have exposure to nesting materials early they are less likely to consume the nesting materials, which is more likely in pet rats. The material allows for good nest weaving and […] is very affordable.” |
| Suitable materials for cage construction must be used | **Important features identified in survey 1 & 2:**  Suitable materials for cage construction | Materials that provide poor ventilation or are difficult to clean are not advised (e.g. vivariums/wooden floors are not advisable). Cages with plastic solid flooring and un-coated metal bars are ideal to avoid injuries, for ease of cleaning, and for ventilation. | **Survey 2, Q3 & Q14 - What sort of cage would provide the best ventilation? & What sort of cage would provide the worst ventilation?**  Open bars (e.g. wire/mesh) – 12 *(Included)*  Not fully enclosed (e.g. avoiding glass/plastic tanks or vivariums) – 15 *(Included)*  Location in room important/avoid drafts – 5 *(Included under ‘The location of the cage must be suitable’)*  **Survey 2, Q8 & Q18 - What would be the best material(s) for the cage construction, including the flooring? & What would be the worst material for the cage construction, including the flooring?**  Metal (good) – 12 *(Included)*  Plastic (good) – 10 *(Included)*  Avoid wire/slatted flooring – 8 *(Included)*  Wood (bad) – 7 (*Not included – only good examples are provided)*  Easy to clean/hygienic – 6 *(Included)*  Plastic (bad) – 6 (*Not included – only good examples are provided)*  Bars for walls (good) – 5 *(Included)*  Chewable materials should be avoided – 5 (*Included under ‘The cage must be designed to reduce the risk of injury’)*  Glass (bad) – 3 (*Not included – only good examples are provided)*  Non-toxic – 3 (*Included under ‘The cage must be designed to reduce the risk of injury’)*  Wood (good) – 2 (*Not included – more popular examples given)*  Materials that cause injuries (e.g. pododermatitis) should be avoided – 2 (*Included under ‘The cage must be designed to reduce the risk of injury’)*  Metal (bad) – 2 (*Not included – conflict with recommendation for metal)*  Ventilation (e.g. not solid sides) – 2 (*Included)*  Avoid cold materials for flooring – 1 (*Partially included – recommendation for bedding substrate to cover entire floor under ‘Rats must be provided with suitable bedding substrates’)*  Allows climbing – 1 *(Included under ‘Rats must be provided with opportunities to exercise’)*  Provides shelter from overhead lighting – 1 *(Included under ‘The location of the cage must be suitable’)* |
| The cage must be designed to reduce the risk of injury | **Important features identified in survey 1 & 2:**  Injury prevention | To avoid injuries: wire flooring should be avoided, soft and wide fall breakers (e.g. hammocks) should be provided, the cage should be monitored for sharp edges from chewed plastic, and any materials that could cause constriction injuries or can catch claws or teeth (e.g. loose weave long fibre fabrics/fluffy bedding/metal chains) should be avoided. Likewise, materials that could pose problems if ingested should be avoided, including rubber objects. Metal bars on the cage must not be coated to avoid metal toxicosis. Wooden items provide gnawing opportunities to avoid overgrown teeth. | **Survey 2, Q17 & Q19 - What cage features could potentially lead to injury? & What cage features could potentially lead to aggression?**  Sufficient space – 9 (*Included under ‘Rats must be provided with sufficient horizontal space’ and ‘Rats must be provided with sufficient vertical space’*)  Sufficient refuge areas – 7 (*Included under ‘Rats must be provided with refuge areas’*)  Sufficient resources/feeding areas/drinking areas – 6 *(Included under ‘Rats must be provided with multiple water bottles/bowls’ & Rats must be provided with multiple food bowls/feeding areas’)*  Use of fall breakers – 6 *(Included)*  Sufficient enrichment items – 5 *(Included under ‘Rats must be provided with a complex environment that includes multiple tiers/levels’)*  Sharp edges (e.g. from chewed plastic) need to be avoided – 5 *(Included)*  Unsafe wheels (e.g., open-wired/slatted or too small*) should not be used – 4 *(Included under ‘Rats must be provided with opportunities to exercise’) **One expert gave a specific recommendation of wheels needing to be >16inches.  Unsuitable cage materials need to be avoided (e.g. wire/slatted flooring that causes pododermatitis/traps feet) – 4 *(Included)*  Materials that could cause injury/death through ingestion should be avoided (e.g. lead/zinc/rubber/plastic) – 3 *(Included)*  Materials that could cause constriction injuries (e.g. chain of metal/bedding) should be avoided – 2 *(Included)*  Sufficiently regular cleaning – 1 *(Partially included under ‘Rats must be provided with suitable bedding substrates’*)  Sufficient ventilation – 1 *(Included under ‘Suitable materials for cage construction must be used’)*  Unsafe substrates (e.g. causes respiratory disease) should be avoided – 1 *(Included under ‘Rats must be provided with suitable bedding substrates’)*  **Survey 2, Q17**  Wood – 13 *(Included)*  Cardboard – 2 (*Not included – more popular examples given)*  Avoid rubber – 1 *(Included)*  Animal bones - 1 (*Not included – more popular examples given)*  Hard-shelled nuts - 1(*Not included – more popular examples given)*  Dog biscuit - 1 (*Not included – more popular examples given)*  Rope – 1 (*Not included – more popular examples given)*  **Online discussion:**  “*Opportunities to gnaw should be higher or incorporated into injury prevention”*  *“I would potentially add in that metal bars on cages should not really be coated (as with bird cages) as chewing can cause metal toxicosis.”*  *“Fall breakers - can we specify that these should be soft or wide? I can imagine some sad endings if people are trying to use branches or ladders as fall breakers.”*  *“Injury prevention - can we include loose weave, long fibre fabrics that can catch claws or teeth? Also, we've recently done a survey about ferret enrichment, and there were a lot of reported injuries due to ingestion or chewing on rubber objects, such as runner balls and kongs, so I wonder if this is the same for rats.”* |
| The location of the cage must be suitable | **Online discussion**  “do we need to suggest where to put the enclosure in a room, e.g., not next to a music system or in a room that doesn't get to hot in the Summer?” | The cage must be located in an area that is not draughty or too cold, that does not get too hot, is not too bright (e.g. not directly below a light), and that is not near any sources of loud noise including ultrasonic noises (e.g. next to a TV, music system, electronic equipment) or other stressors (e.g. predator species like cats). | **Survey 2, Q3 & Q14 - What sort of cage would provide the best ventilation? & What sort of cage would provide the worst ventilation?**  Location in room important/avoid drafts – 5  **Survey 2, Q8 & Q18 - What would be the best material(s) for the cage construction, including the flooring? & What would be the worst material for the cage construction, including the flooring?**  Provides shelter from overhead lighting – 1  **Online discussion**  “do we need to suggest where to put the enclosure in a room, e.g., not next to a music system or in a room that doesn't get to hot in the Summer?”  “should also mention that at least some of the cage, e.g. the refuges, should be protected from draughts.”  “I was also thinking about placement, not too hot / cold / bright and also away from other animals or noise that might cause them stress.” |
